# Structural Basis of *Clostridium perfringens* Enterotoxin Activation and Oligomerization by Trypsin

**DOI:** 10.3390/toxins15110637

**Published:** 2023-10-31

**Authors:** Chinemerem P. Ogbu, Srajan Kapoor, Alex J. Vecchio

**Affiliations:** Department of Structural Biology, University at Buffalo, The State University of New York, Buffalo, NY 14203, USA; cpogbu@buffalo.edu (C.P.O.); kapoorsrajan@gmail.com (S.K.)

**Keywords:** *Clostridium perfringens* enterotoxin, claudins, tight junctions, gastrointestinal epithelia, cell/cell interactions, membrane proteins, bacterial cytotoxicity, food poisoning

## Abstract

Clostridium perfringens enterotoxin (CpE) is a β-pore forming toxin that disrupts gastrointestinal homeostasis in mammals by binding membrane protein receptors called claudins. Although structures of CpE fragments bound to claudins have been determined, the mechanisms that trigger CpE activation and oligomerization that lead to the formation of cytotoxic β-pores remain undetermined. Proteolysis of CpE in the gut by trypsin has been shown to play a role in this and subsequent cytotoxicity processes. Here, we report solution structures of full-length and trypsinized CpE using small-angle X-ray scattering (SAXS) and crystal structures of trypsinized CpE and its C-terminal claudin-binding domain (cCpE) using X-ray crystallography. Mass spectrometry and SAXS uncover that removal of the CpE N-terminus by trypsin alters the CpE structure to expose areas that are normally unexposed. Crystal structures of trypsinized CpE and cCpE reveal unique dimer interfaces that could serve as oligomerization sites. Moreover, comparisons of these structures to existing ones predict the functional implications of oligomerization in the contexts of cell receptor binding and β-pore formation. This study sheds light on trypsin’s role in altering CpE structure to activate its function via inducing oligomerization on its path toward cytotoxic β-pore formation. Its findings can incite new approaches to inhibit CpE-based cytotoxicity with oligomer-disrupting therapeutics.

## 1. Introduction

*Clostridium perfringens* is a Gram-positive spore-forming bacterium that causes enteric and histotoxic diseases in humans and domesticated animals [1]. Upon sporulation, type F strains produce an enterotoxin, CpE, which causes the second most common bacterial food-borne illness in the United States and other developed countries [2]. CpE is a 35 kDa protein with 319 amino acids and a two-domain structure that resembles other β-pore forming toxins [3,4]. Domain I corresponds to the N-terminal region (nCpE) and comprises amino acids 1–197, while domain II constitutes the C-terminal region (cCpE) comprising amino acids 198–319. Functional characterization of CpE revealed that nCpE functions in cytotoxicity and β-pore formation, while cCpE is the receptor binding domain for claudins, a family of ~25 kDa integral membrane proteins that reside at epithelial tight junctions in the mammalian gut [5,6].

The proposed sequence of events that lead to CpE cytotoxicity involves 35 kDa CpE binding to ~50 kDa claudin dimers to form a 90 kDa “small complex”. This small complex hexamerizes and associates with non-receptor claudins and/or occludin to form a ~450 kDa complex cytotoxic β-pore [7]. Like other β-pore-forming toxins, nCpE would form a membrane-penetrating β-barrel, while cCpE tethers the complex to apical cell membranes via claudin interactions. CpE β-pore triggers calcium influx, resulting in apoptosis or oncosis depending on cellular concentration [8]. It has also been shown that cCpE removes claudins from tight junctions in the absence of nCpE, implying that both domains can alter epithelial permeability in unique ways (although nCpE without a cCpE domain cannot function) [9].

In the mammalian gut, spore-formed *C. perfringens* secreted CpE is susceptible to degradation by proteases. Previous studies have shown that engineered removal of the first 44 amino acids of CpE does not decrease but instead activates cytotoxicity in vivo and that deletion beyond this point abolishes cytotoxicity [10]. Other studies showed that proteolytic cleavage of CpE by trypsin triples its activity due to a loss of a 4 kDa peptide and that this cleavage occurs at CpE’s N-terminus between residues Lys15/Glu16 and Lys25/Thr26 [11]. Crystal structures of full-length CpE show that the first 35 residues are not observed, indicating that CpE’s N-terminus is likely unstructured [12,13]. Subsequent structures of CpE with its first 37 residues removed have been determined, but whether this truncation produces the same increase in activity as trypsin has not been established. The mechanism of CpE activation by trypsin and the role of the N-terminus in the CpE function each lacked atomic-level understanding.

While previous X-ray crystal structures have revealed the interactions between cCpE and claudins that are required for selectivity and high-affinity binding, they have not elucidated the process of CpE oligomerization that occurs once bound to claudins that ultimately leads to the formation of cytotoxic β-pores. We thus sought to clarify the effect of trypsinization and the role of the CpE N-terminus in CpE activation and oligomerization through a structural biology lens. To accomplish this, we used mass spectrometry to pinpoint the trypsin proteolytic site(s) on CpE, small-angle X-ray scattering to determine structures of full-length wild-type CpE and trypsinized CpE, and X-ray crystallography to determine high-resolution structures of trypsinized CpE and wild-type cCpE in two new crystal forms. Moreover, we modeled the structures of physiologically relevant CpE oligomers and their conformational changes that lead to cytotoxic β-pores. Our results provide a mechanism for CpE activation by trypsin, and a structural basis for trypsin-induced oligomerization of CpE may yield cytotoxic β-pores.

## 2. Results

### 2.1. Effect of Trypsin on CpE’s N-Terminus

**Figure 1:**
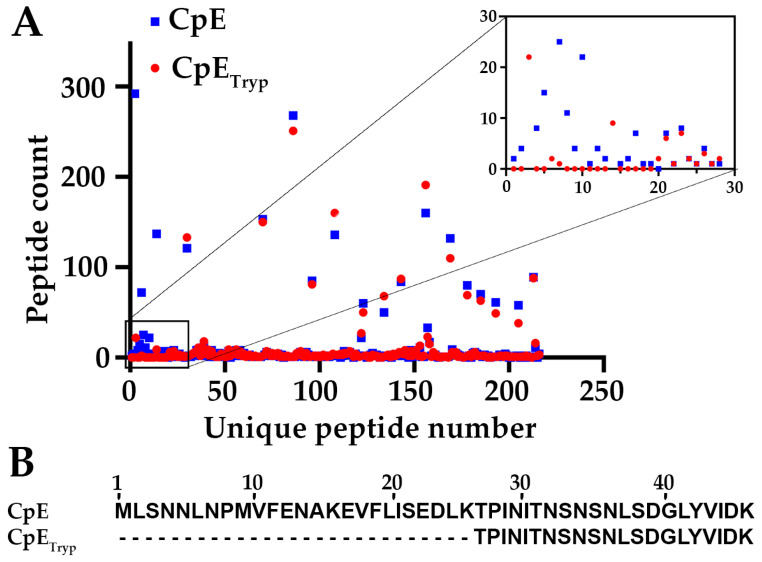
**Mass Spectrometry analysis of CpE and CpE_Tryp_.** (**A**) Graphical representation of the quantified unique peptides measured for CpE (blue square) and CpE_Tryp_ (red circle). Inset shows peptide counts with the most differences between CpE and CpE_Tryp_. (**B**) Sequence alignment of the major peptides of the N-terminus between CpE and CpE_Tryp_.

After expressing and purifying CpE using established protocols, we sought to determine trypsin’s cleavage site(s). We treated full-length CpE with bovine trypsin immobilized on agarose beads, ran SDS-PAGE, and then analyzed excised bands for trypsinized CpE (CpE_Tryp_) and untreated full-length CpE using trypsin-initiated mass spectrometry (MS). MS results revealed that CpE and CpE_Tryp_ had 93% sequence coverage. For untreated CpE, 2761 spectra include 200 unique peptides, whereas, for CpE_Tryp_, 2214 spectra include 195 unique peptides. Comparative analysis of the spectra showed that the total number of peptides differed. We quantified peptide counts identified for each sample and assigned numbers ranging from 1 to 220 for each unique peptide identified (Figure 1A). Table S1 provides a list of all identified peptides, their unique peptide number, and peptide count. Differences between CpE and CpE_Tryp_ peptides appeared within the first 30 peptide numbers, which corresponded to the first 46 amino acids within the N-terminus (Table S1). Mapping the peptides to the CpE sequence showed that the missing peptides in CpE_Tryp_ represent the first 25 amino acids (Figure 1B). The terminal cut site of trypsin sits between Lys25 and Thr26. Our MS results revealed that CpE_Tryp_ N-terminus begins at Thr26 and is thus 25 amino acids shorter than wild-type CpE (Figure 1B). Computational predictions of molecular mass suggested that this truncation would constitute a ~3 kDa loss. This analysis became the basis for structural studies.

### 2.2. Biophysical Analysis of CpE and CpE_Tryp_

**Figure 2:**
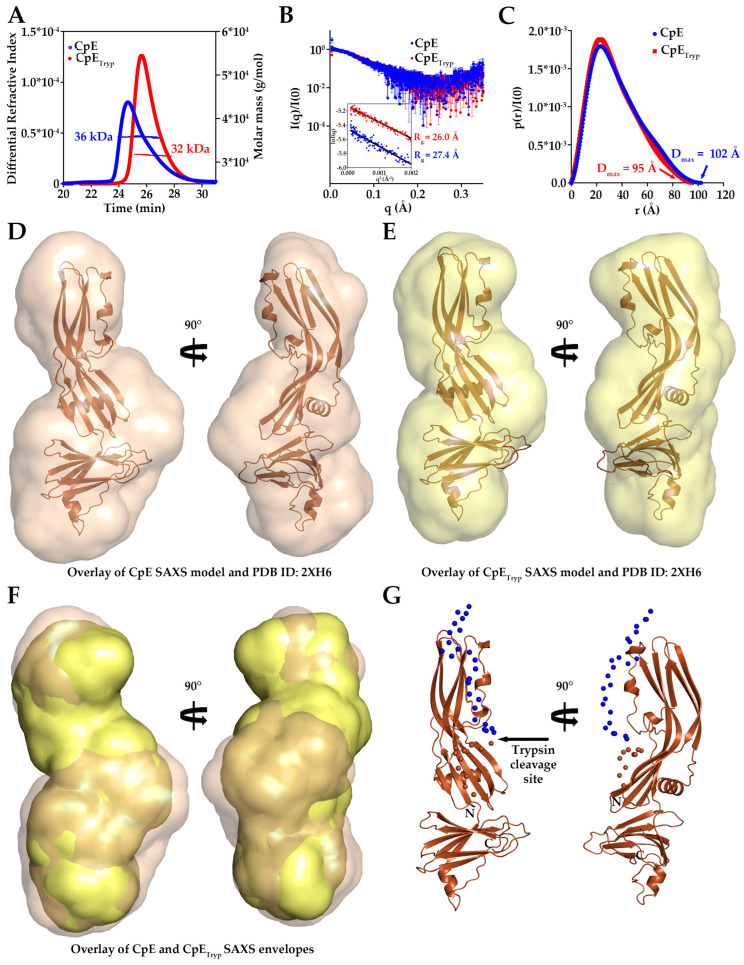
**Biophysical Parameters and Solution Structures of CpE.** (**A**) SEC–MALS molar mass determination for CpE (blue) and CpE_Tryp_ (red). (**B**) I(q) vs. q as log-linear plots showing the scattering profile with inset showing the Guinier fits for qR_g_ <1.3. The R_g_ of CpE (blue) and CpE_Tryp_ (red) is quantified. (**C**) p(r) function plot obtained by indirect Fourier transform of I(q). D_max_ calculated for CpE (blue) and for CpE_Tryp_ (red) are shown. (**D**) Space–filled SAXS envelope of CpE (tan) from GASBOR with modeled CpE crystal structure (PDB ID: 2XH6) using CIFSUP. (**E**) Space–filled SAXS envelope of CpE_Tryp_ (yellow) generated as in D. (**F**) Overlay of SAXS densities of CpE (tan) and CpE_Tryp_ (yellow) to highlight size and shape differences. (**G**) Ensemble Optimization Method prediction for the position of N–terminal residues 1–25 (spheres, blue) and 26–37 (spheres, brown) of CpE (cartoon, brown). The site of trypsin cleavage is highlighted.

To decipher the biophysical consequence of N-terminal truncation of CpE by trypsin, we used size-exclusion chromatography-multi-angle light scattering (SEC-MALS) and small-angle X-ray scattering (SAXS) to determine absolute molecular mass and to approximate shapes of CpE and CpE_Tryp_. SEC-MALS results showed that the mass of CpE is estimated to be 36 kDa, while for CpE_Tryp_, it is 32 kDa (Figure 2A). This loss of ~4 kDa of mass agreed with computational predictions of ~3 kDa and a previous study by Granum et al. [11,14]. After subtracting the buffer, we performed the SAXS analysis using the frames corresponding to the protein peak (Figure S1A). SAXS analysis revealed that the overall size of CpE, as indicated by the radius of gyration (Rg) from the Guinier plot, was 27.4 ± 0.4 Å, and for CpE_Tryp_ it was 26.0 ± 0.2 Å (Figure 2B). Pair distance distribution analysis (p(r)), which shows the internal distances within the proteins, revealed a maximum particle dimension (D_max_) of 102 Å for CpE and 95 Å for CpE_Tryp_ (Figure 2C). Both proteins show a similar degree of flexibility and folding, as seen in the bell-shaped profile with a defined maximum in the dimensionless Kratky plot (Figure S1B). The molecular weight estimations from SAXS, 37.8 kDa and 30.0 kDa for CpE and CpE_Tryp_, respectively, agree with those from SEC-MALS and computational analyses (Table S2). In sum, biophysical analyses of CpE and CpE_Tryp_ suggest that the samples are monomeric but differ in mass and shape.

### 2.3. Solution Structures of CpE and CpE_Tryp_

Using SAXS, we determined low-resolution solution structures of CpE and CpE_Tryp_ to visualize measured size and shape differences. At the concentrations analyzed for SAXS, no significant amount of dimer or larger oligomers was detected (Figure 2A,B). Ab initio bead models were built, which showed both proteins existed as elongated structures with bulky and narrow ends (Figure 2D,E). The envelope densities for both toxins were fit using an existing crystal structure (PDB ID: 2XH6) that had the terminal 37 residues removed. These structural overlays showed that cCpE aligns with the bulky end of the envelope while nCpE fits within the narrow end of the SAXS envelopes for both enterotoxins. The crystal structure fit, as analyzed by FoXS, showed χ^2^ fits of 1.03 and 0.96 for CpE and CpE_Tryp_, respectively (Figure S1C). However, to achieve the χ^2^ of 1.03, the c_2_ value in FoXS was increased to 4.0, while for CpE_Tryp_, c_2_ was 0.6. Because c_2_ is a parameter that controls the density of water around a molecule during FoXS fitting, the higher c_2_ of CpE suggested that this crystal structure lacked residues that were present in the SAXS data, whereas this was not true for CpE_Tryp_. The solution structures of individual CpEs revealed differences that we further investigated.

We superpositioned the SAXS densities for both CpEs, which showed that CpE was longer and wider than CpE_Tryp_, especially in the area that transitions from nCpE to cCpE domains (Figure 2F and Figure S1D). The added bulk on both ends of the CpE envelope explains our observations reported previously, where various biophysical parameters showed that CpE was larger in radius and mass compared to CpE_Tryp_. The bulky end of CpE where cCpE resides is bulkier than the same end of CpE_Tryp_, suggesting that for full-length CpE, its cCpE domain is more dynamic and samples a larger conformational space than the corresponding cCpE from CpE_Tryp_. Added density in the middle of the CpE envelope was harder to discern as it constitutes a portion of nCpE, and we did not expect sub-domains of nCpE to move independently. We hypothesized that this added density could be caused by the presence of the N-terminus. We then predicted the position of the N-terminal residues of the full-length CpE using the Ensemble Optimization Method (EOM). Several resulting structures were obtained, which we filtered based on their agreement with our experimental data, especially D_max_ (Figure S1E and Table S3). Using these criteria, one model had the appropriate D_max_. Visualization of this model revealed the approximate position of the N-terminal 37 residues that fit the CpE SAXS envelope (Figure 2G). The first 25 amino acids were predicted to begin at the narrow end of CpE and extend into the space between the nCpE and cCpE domains, with residues 26–37 occupying this region. This validated that the added bulk of CpE was due to N-terminal presence and dynamics. The solution structures generated by SAXS of CpE and CpE_Tryp_ provided a basis for the effect of trypsinization and the role of the N-terminus in altering CpE structure.

### 2.4. Crystal Structure of CpE_Tryp_ Uncovers Dimer Interfaces

**Figure 3:**
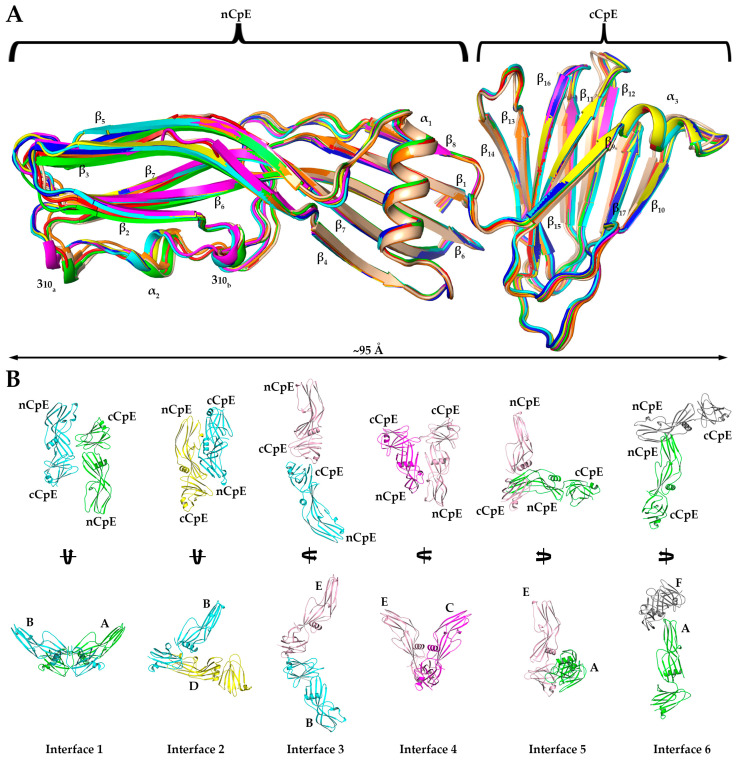
**Crystal Structure of CpE_Tryp_.** (**A**) Structural alignment of eight CpE_Tryp_ molecules observed in our structure with each secondary structural element and domain labeled accordingly. (**B**) The six homo–dimer interaction interfaces found in the asymmetric unit of the CpE_Tryp_ crystal structure. Each monomer is colored uniquely, and specific chains and domains are labeled accordingly. Interfaces are numbered according to their predicted strength of interactions (left to right, from strongest to weakest), as assessed by PDBePISA. Interface 1 is between chains A (green) and B (cyan) with an anti-parallel orientation. Interface 2 is between B (cyan) and D (yellow), with compact “<” shape. Interface 3 is between B (cyan) and E (salmon), forming an extended “<” shape. Interface 4 is between C (magenta) and E (salmon), with ”U” shape. Interface 5 between A (green) and E (salmon) forms an “L” shape. Interface 6 between A (green) and F (grey) forms a “T” shape.

As structures of full-length and non-proteolyzed but Δ37 truncated CpE existed but were not significantly different structurally, we intended to gain deeper insights into the effect of trypsin on CpE by obtaining higher resolution structures of CpE_Tryp_ than afforded by SAXS. We determined a 2.3 Å crystal structure of CpE_Tryp_ after obtaining crystals that belonged to space group P 4_3_ 2 2 with unit cell dimensions and angles of 200.33, 200.33, and 254.78 Å and 90°, 90°, and 90°, respectively (Table S4). The determined structure’s asymmetric unit was found to contain eight CpE_Tryp_ molecules (Figure S2A). This crystal packing is a unique feature of the structure, whereas other CpE crystal structures contained one, two, three, six, or sixteen molecules in the asymmetric unit that came from packing in space groups P 2_1_ 3 or C 1 2 1 (Figure S2B–D). The overall CpE_Tryp_ structure resembled CpE, wherein it comprises 17 β-strands arranged in five β-sheets, three α-helices, and two 3_10_-helices (Figure 3A). No secondary or tertiary structural features were significantly altered by trypsin. Yet, despite structural similarities, the existence of eight CpE_Tryp_ molecules in the asymmetric unit exhibited novel quaternary structure and allowed us to analyze CpE_Tryp’s_ non-crystallographic oligomeric surfaces.

Although no higher-order oligomers of CpE_Tryp_ were present, we observed 10 unique dimer interfaces in the crystal structure between the eight CpE_Tryp_ chains. Of these, six existed as non-crystallographic symmetry-induced interactions within the asymmetric unit (Figure 3B). We classified these six interfaces from strongest to weakest by estimating the interface areas (Å^2^), solvation-free energy gain upon interface formation (Δ^i^G, kcal/mol), and number of interfacing atoms (N_Atom_) and residues (N_Residue_) involved in the formation of each interface using Protein Interfaces, Surfaces, and Assemblies (PDBePISA) (Table 1) [15]. Interface 1 is the strongest interface, formed by chains A and B, and is structurally equivalent to the interface formed by G and H. At interface 1, the two chains interact using the β_4_ strand and loop region between α_1_ and β_4_ of one subunit associating with the loop regions between β_14_–β_15_ and β_16_–β_17_ of another subunit. Here, cCpE/nCpE interactions are used to form an anti-parallel “I-I” shaped dimer. The A/B interface 1 is stabilized by six hydrogen bonds, while the equivalent for G/H has three. Interface 2 is formed between chains B and D and is equivalent to chain A/C and E/G interfaces. This dimer orients in an anti-parallel manner but forms a compact “<” shape where cCpE/nCpE interactions between distinct monomers drive association. The interface involves the α_1_ helix and β_8_–β_9_ loop of one chain interacting with the α_2_ helix of another chain and is stabilized by 6–7 hydrogen bonds and a salt bridge between Asp175 and His241. Interface 3 is observed between chains B/E and C/H and is formed from β_12_, β_15_, and β_17_ residues of one subunit interacting with β_10_ and β_17_ loop residues from another and uses cCpE/cCpE interactions. The two cCpEs contact one another in a tail-to-tail orientation to form an extended “<” shaped dimer. This interface contains five hydrogen bonds. Interface 4 connects chains C to E and employs the loops between α_1_ and β_4_ and β_11_ and β_12_, interacting with their equivalent regions on another subunit. Here, cCpE/cCpE interactions drive self-association, as does a portion of nCpE, forming a “U” shaped dimer. Two hydrogen bonds exist here. For interface 5, A/E and C/G chains associate through loop β_13_ and β_14_ contacting the β_7_ strand and α_2_ helix. This dimer is anti-parallel oriented driven by cCpE/nCpE interactions that form an “L” shape. This interface has 3–4 hydrogen bonds, depending on chains. Interface 6, the weakest interface, is formed via chains A and F and is the only interface driven solely by nCpE/nCpE interactions. Here, the β_2_–β_3_ and β_5_–β_6_ loops of the A monomer interact with a cleft formed behind the unstructured linker between the nCpE and cCpE domains of F. This dimer is “T” shaped and has no hydrogen bonds but does associate via non-polar interactions. The crystal structure of CpE_Tryp_ revealed six novel dimer interfaces, with five of the most stable employing the cCpE domain to direct homodimerization. We subsequently investigated cCpE/cCpE dimers further.

### 2.5. New Crystal Forms of Dimerized cCpE

**Figure 4:**
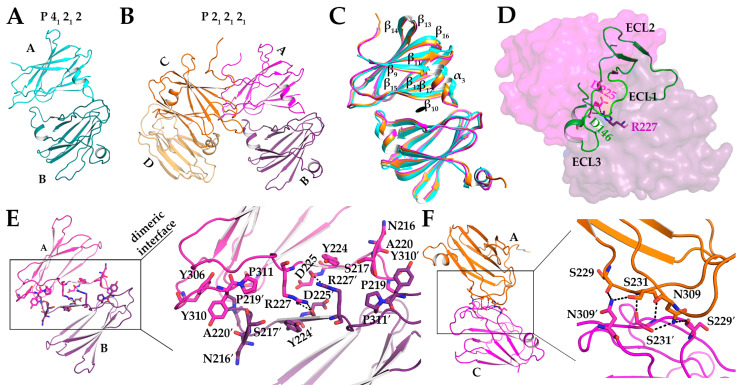
**Homodimer Interfaces in cCpE Structures.** (**A**) Chain A (cyan) and B (teal) of the P 4_1_ 2_1_ 2 crystal dimer. (**B**) Chains A (magenta), B (purple), C (orange), and D (light orange) of the P 2_1_ 2_1_ 2_1_ crystal dimer of dimers. (**C**) Alignment of three cCpE homodimers depicted in A and B. (**D**) Superposition of cCpE from PBD ID: 6OV2 onto chain B of the cCpE dimer. Claudin (green) binds to chain B of cCpE (purple) in the region where chain A (magenta) does. (**E**) Residues involved in the dimer interface common to both structures with each chain colored as in B. (**F**) Residues involved in the unique dimer interface between chains A and C from the P2_1_ 2_1_ 2_1_ structure with each chain colored as in B. Hydrogen bonds are depicted as dashed lines (black).

As only one structure of cCpE existed, and we found that this domain drove the five major dimer interfaces observed in the structure of CpE_Tryp_, we sought to structurally characterize cCpE further to assess whether cCpE/cCpE dimers were possible *in crystallo*, and if so, if they employed identical surfaces to CpE_Tryp_. The existing cCpE structure, PDB ID: 2QUO, is composed of residues 194–319 and crystallized in space group P 2_1_ 2_1_ 2_1_ with one cCpE molecule in the asymmetric unit—thus, no non-crystallographic dimers were observed [16]. We determined crystal structures of cCpE comprised residues 194–319 in two different crystal forms—P 4_1_ 2_1_ 2 and P 2_1_ 2_1_ 2_1_—the latter being the same space group as 2QUO (Table S4). The P 4_1_ 2_1_ 2 structure was resolved to 1.6 Å, had unit cell dimensions and angles of 65.12, 65.12, and 130.77 Å and 90, 90, and 90°, respectively, and contained a dimer in the asymmetric unit (Figure 4A). The P 2_1_ 2_1_ 2_1_ structure was resolved to 1.4 Å, had unit cell dimensions and angles of 65.93, 65.93, and 136.98 Å and 90, 90, and 90°, respectively, and had a dimer of dimers in the asymmetric unit (Figure 4B). Overall, both crystal forms showed that the cCpE tertiary structure consisted of a nine-strand β-sandwich with a short α-helical segment between Leu211 and Ser217 (α_3_). Structural alignment of the cCpE monomers from both crystal forms revealed that they share this same topology. This is verified by root mean square deviations (RMSDs) between Cαs of individual monomers from both structures ranging between 0.25 and 0.37 Å, indicating high structural convergence, although minor deviations exist in loop regions that connect secondary structural elements. Despite these similarities in secondary and tertiary structures, the quaternary structures of these two structures diverged from 2QUO and from each other, revealing two novel dimer interfaces.

Our two crystal structures contain three homodimers in their respective asymmetric units, two in the P 2_1_ 2_1_ 2_1_ space group (Figure 4A,B). Structural alignments of the Cαs between the three dimers revealed RMSDs that range from 0.93 to 1.2 Å (Figure 4C). From a global standpoint, the three dimers are related by C2 symmetry and are formed by the same type of interaction, where the turn between the α_3_ helix and β_10_ tucks into the cleft formed between β_16_ and β_17_ of the opposing monomer (Figure 4C). Interestingly, this α_3_–β_10_ loop binds in the region where the second extracellular segment loop of claudins binds cCpE within the β_16_–β_17_ cleft, also known as the claudin pocket (Figure 4D) [4]. Analysis of this homodimeric surface showed that ~10% of the total surface area of each monomer is involved in dimerization. Although the homodimers shared this particular interface, differences arose that explained the divergence in packing that resulted in alternate space group assignments.

In the P 4_1_ 2_1_ 2 single homodimer crystal form, the dimer interface is stabilized by three hydrogen bonds and one electrostatic bond. The carbonyl oxygen of Asp225 forms a hydrogen bond with the amino groups of Arg227 for both chains A and B (Figure 4E). Arg277 of chain A forms a salt bridge with Asp225. Lys257 on chain A also forms a hydrogen bond with Ala220 chain B. Pro219 and Ala220 on chain A penetrate a hydrophobic pocket containing Ile258, Tyr306, and Tyr310 on chain B and forms an aromatic-cis-Pro interaction with Tyr310 (Figure 4E). Two similar dimers exist in the P 2_1_ 2_1_ 2_1_ crystal, formed from A/B and C/D interactions. The A/B dimer is stabilized by four hydrogen bonds and the C/D by three. For A/B, Asp218 forms a hydrogen bond with Ser313 and Asp225 hydrogen bonds with itself on both chains and with Arg227. Arg227 of chain A then forms a hydrogen bond with Tyr244. For C/ D, Asp225 forms a hydrogen bond with Arg227. The hydrogen bond between Arg227 and Asp225 is common to all three dimers (Figure 4E). Arg227 of cCpE has been shown to form a salt bridge with Asp146 of claudin-9, so an intermolecular salt bridge between cCpEs can be expected [17]. In full, although the dimer interface described above is commonly used three times between the two structures, minute differences are present that result in localized changes that ultimately influence the global packing of cCpE molecules.

The P 2_1_ 2_1_ 2_1_ crystal structure has one unique dimer interface between chains A and C. This dimer employs six hydrogen bonds (Figure 4F). Here, Asn309 of chain A forms hydrogen bonds with Ser229, Ser231, and Asn309 of chain C, and Ser229 of chain A forms hydrogen bonds with Asn309 of chain C. No direct interaction is seen between chain B and chain D. This unique dimer explains the alternate crystal packing and space groups between cCpE structures. Ultimately, the cCpE/cCpE interfaces and interactions found from structures of the cCpE domain alone did not provide further insights into the homo-oligomerization of CpE because the CpE_Tryp_ structure did not possess homologous interfaces. We, therefore, sought to discern how nCpE’s presence helps to uniquely organize CpE oligomers by providing interfaces not attainable by cCpE alone.

### 2.6. Models of Claudin-Bound CpE Oligomers Uncover Relevance of CpE Structures

We next aligned the Cαs from the eight monomers of CpE_Tryp_ to previously determined CpE structures to visualize the potential effect of trypsin (Figure S3). We found that all CpE monomers aligned well with RMSDs between 0.175 and 0.877 Å. Differences between structures occurred at loops that connect secondary structural elements, which likely result from crystal packing. Despite numerous structures of CpE having been determined by X-ray crystallography, some that revealed trimers in the asymmetric unit or via crystallographic symmetry and hexamers generated from similar homologous toxins, the biological relevance of CpE trimers or hexamers has not been established in the context of claudin binding. We, therefore, used our CpE_Tryp_ structure and previously determined structures to establish the significance of CpE homo-oligomerization in relation to the plasma membrane, where the cytotoxic pore forms due to high-affinity interactions with claudins.

**Figure 5:**
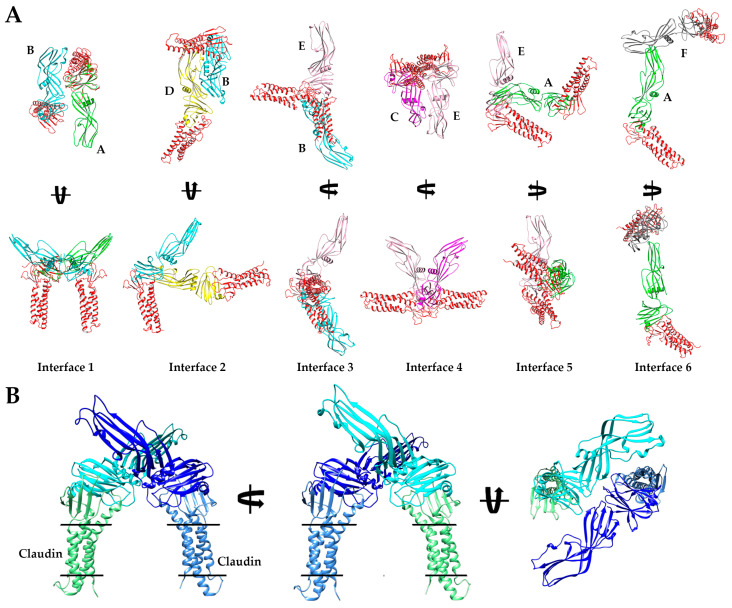
**Models of Claudin-bound CpE Dimers.** (**A**) Structural representation of the six interfaces from CpE_Tryp_ as shown and colored in Figure 3B. Here, the cCpE from PDB ID: 6OV2 is superposed with the cCpE from each CpE_Tryp_ to visualize the position of bound claudin (red). Protein chains are labeled from A–F according to the way they are represented in the PDB ID: 8U5F. (**B**) Three-dimensional orientation of the modeled claudin-bound Interface 1 dimer with one claudin/CpE complex colored two shades of blue or green.

We next assessed whether claudins were organized perpendicular to and within the same membrane when bound to CpE_Tryp_ dimers, which would indicate a physiological pose. Upon superposition of cCpE from the structure of it bound to claudin-9 (PDB ID: 6OV2) onto the cCpE domain of all six dimer interfaces of CpE_Tryp_, we observed that for the A/B and G/H dimers (Interface 1, the most stable), both claudins had these properties, whereas claudins bound to the other five interfaces lacked perpendicular orientation to the membrane or clashed significantly with other molecules in the complex (Figure 5A). Further analysis of claudin-bound Interface 1 dimers showed no steric clashes and that each monomer is related to the other by a rotation and translation about a C2 axis (Figure 5B). We searched the PDB for similar interfaces by examining 170,428 structures and 4,348,532 interfaces. Of these, only seven similar interfaces were found. All these interfaces came from other CpE structures, specifically PDB IDs: 2YHJ, 2XH6, 3AM2, 3ZIW, 3ZIX, 3ZJ3, and 4P5H. We evaluated these seven interfaces and found none possessed an orientation where cCpE-bound claudins were perpendicular to the membrane plane (Figure S4). We also analyzed the trimer made from crystal symmetry and found that if claudins bound to it, they would not reside within the same membrane either (Figure S4C). The results of these analyses showed that Interface 1 from CpE_Tryp_ was structurally unique compared to every protein interface across the PDB and even compared to other structures of CpE. This showed that the specific removal of the first 25 residues by trypsin leads to the formation of at least one novel homo-oligomeric state. We next aimed to assess if CpE_Tryp_ monomers could form higher-order oligomers and if Interface 1 could act as a building block to such oligomers.

### 2.7. CpE Interface 1′s Use in Higher Order Oligomerization and β-Pore Formation

**Figure 6:**
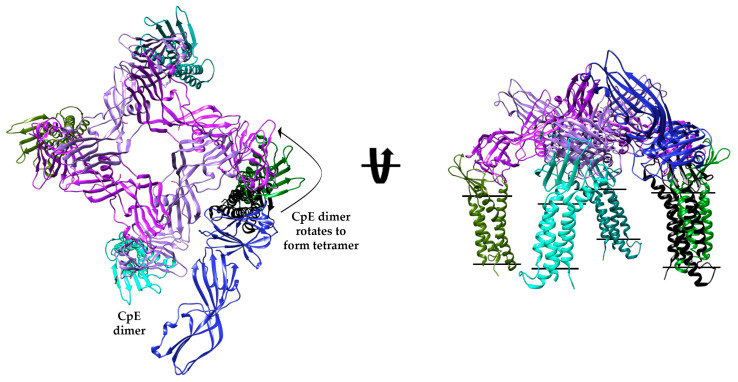
**Model of a Claudin-bound CpE Tetramer and Mechanism of Interface 1 Dimer Incorporation.** Predicted model of a biologically relevant CpE_Tryp_ tetramer (purple and plum) from GalaxyHomomer with bound claudins (various shades of green). The tetramer is overlaid with Interface 1 dimer bound to claudin. While one claudin/CpEs complex overlays perfectly, the other claudin/CpE (black/blue) complex sits outside the central pore and, if positioned as the tetramer, should overlay with the green claudin.

Because CpEs’ cytotoxic β-pore is believed to assemble as a hexamer, we intended to determine if our Interface 1 homodimer could act as a building block for higher order CpE oligomers and whether those oligomers bound properly oriented claudins. We used chain A from CpE_Tryp_ to predict homo-oligomerization, searching independently for dimers, tetramers, hexamers, and octamers—multiples of two to distinguish Interface 1 dimers relevance. This exercise produced five models for each oligomer queried (20 total). We analyzed all 20 oligomers by superposing 6OV2 onto the cCpE domain of all CpE chains as before and found that no predicted dimers, hexamers, or octamers possessed claudins that resided perpendicular within membranes. However, of the five predicted tetramers, one had characteristics of biological relevance, exhibiting all four bound claudins residing in the membrane, the hypothetical pore-forming helix (α_1_) of nCpE positioned down toward the outer membrane leaflet, and Asp48 pointing toward the center cavity formed at the tetramer interface (Figure 6). To visualize how the Interface 1 dimer fits within this tetramer, we superposed chain A from CpE_Tryp_ onto the tetramer and found that the claudin/CpE from one complex overlayed perfectly onto the corresponding complex found in the claudin-bound tetramer (Figure 6). The chain B claudin/CpE complex, however, had its CpE oriented outside of the central cavity. This causes the claudin to bind near its equivalent claudin bound to the CpE tetramer but not exactly, with distances between claudins that range from 18 to 39 Å for equivalent atoms. This claudin/CpE complex would thus require a translation and rotation of either −90° or +270° to avoid clashes with CpE from complex A to form the tetrameric arrangement. We modeled the conformational change that two Interface 1 dimers may take to form the tetramer by generating structural morphs (Movies S1 and S2). The morphs depict how two Interface 1 dimers could tetramerize through a −90° rotation of two claudin/CpE complexes while the other two remain static to form the hypothetical tetramer. As a result of this modeling, we hypothesize that the Interface 1 homodimer observed in the CpE_Tryp_ structure may have biological relevance toward forming higher-order oligomers on CpE’s path toward the formation of a cytotoxic β-pore and that trypsin facilitates this process through the removal of 25 of CpE’s N-terminal residues.

## 3. Discussion

This study’s findings aid our understanding of CpE activity and reveal mechanisms of CpE oligomerization on its path toward cytotoxic β-pore formation. First, it confirms the biochemical identity of trypsin-treated CpE using MS and shows the first 25 amino acids of the N-terminus are removed by this protease. This result agrees with the study by Richardson and Granum [14]. The absolute molecular mass measurements and solution structure envelopes generated by SEC-MALS and SAXS of CpE and CpE_Tryp_ provide evidence that the loss of CpE N-terminal residues by trypsin creates a quantitatively smaller massed and more compact protein. We measure a loss of 4 kDa, which also agrees with the previous literature [11]. Our data show that at the concentrations applied to an SEC column, CpE and CpE_Tryp_ are monomeric. In addition, we show that at higher concentrations induced by crystal nucleation, CpE/CpE assembly occurs and is primarily driven by interactions of cCpE with nCpE. In our attempts to crystallize CpE_Tryp_, we set up crystallization experiments of full-length wild-type CpE in parallel using the same screens and protein concentration. We found that CpE yielded considerably fewer crystal hits compared to CpE_Tryp_. This is further evidence that trypsin’s removal of CpE’s N-terminus alters CpE structure to induce protein/protein interactions that are thermodynamically unlikely to occur without removal of the N-terminus.

Crystal structures of CpE_Tryp_ and cCpE demonstrate that cCpE/cCpE dimer interactions are favorable and stable in the absence of nCpE but less favorable when nCpE is present. The two cCpE/cCpE interfaces we observe in the two crystal structures of cCpE are not physiologically relevant due to the approximated positions of claudins when bound to them. These structures, however, provide an insight into the experimental observation that treatment of claudin-expressing cells with cCpE removes claudins from tight junction strands [9]. The major cCpE/cCpE dimer interface is one where each cCpE is anti-parallel oriented 180° and thus would sterically prevent claudin binding. Yet Sonoda et al. observed that cCpE application to an epithelial monolayer removes claudins from tight junctions [9], which suggests that this cCpE/cCpE dimer is a lower affinity interaction than a claudin/cCpE interaction. In solution at pathophysiological concentrations, cCpE is monomeric, like CpE. In vivo, this may translate to cCpE remaining non-self-associated, leaving it free to bind claudins. This is validated by the CpE_Tryp_ structure, which, in the absence of bound claudins and with a nCpE domain present, contains only one cCpE/cCpE-driven interface. This Interface 3 is not structurally identical to the anti-parallel cCpE/cCpE interface, yet it cannot bind two claudins simultaneously. These observations, taken together with our characterization of six CpE/CpE interfaces in the CpE_Tryp_ structure, show that although cCpE drives CpE self-assembly, the resulting assemblies do not occlude the claudin pocket. Further, we observe that nCpE/nCpE interfaces are disfavored. Thus, we show that four of the six most prevalent interfaces employed for CpE assembly do not employ the claudin pocket of cCpE nor nCpE alone for CpE homo-oligomerization. Each CpE extremity is, therefore, free to function in their respective roles of claudin binding (cCpE) or β-pore formation (nCpE). We believe that these results hold physiological significance by showing that each extremity is not employed in CpE homo-oligomerization and that this function is relegated to the region between domains.

Because numerous structures of CpE have been determined, and the structural basis of building a CpE oligomer from a single monomer remained undetermined, we analyzed all CpE structures to discern their biological significance and to establish if our trypsinized CpE structure was structurally distinct. For this, we modeled claudin-bound CpEs based on crystal structures of claudins in complex with cCpE. Crystal-induced versus potentially biologically relevant structures, we argue here, can be more easily discerned this way. Using this premise, we show that no CpE structure determined to date yields a biologically relevant claudin-bound CpE complex because the bound claudins are not organized perpendicular to a hypothetical membrane plane. Other researchers have likely come to these conclusions, as Briggs et al. modeled a hexameric CpE pre-pore not from a structure of CpE but from that of hemagglutinin HA70/HA3 from *Clostridium botulinum* [12]. Although that hexamer may be relevant in CpE function, we show that the trimer observed in many CpE structures cannot be membrane-active and is likely not formed during CpE cytotoxicity because the claudins the trimer binds cannot be membrane inserted. For it to be relevant, it would need to be considerably rearranged before binding claudins, yet there is no evidence for CpE oligomers pre-forming before claudin binding. In our analysis of the eight monomers of CpE_Tryp_, we find homo-dimers but no larger oligomers. However, we show that the Interface 1 homo-dimers consisting of chain A/B and G/H represent a novel interface amongst the entire PDB and is the most prevalent, energetically favorable, stable, and has the greatest interface area of the six interfaces we observed. We also show that claudins bound to Interface 1 dimers would be positioned properly within membranes. We thus propose that the Interface 1 dimer, which could form in the presence or absence of claudins, is biologically significant and could be the basic subunit of larger CpE assemblies.

Lastly, we attempt to discern whether the unique Interface 1 homo-dimer could be a building block for higher-order CpE oligomers. Using only a CpE_Tryp_ monomer as input, we describe via computational predictions that all generated homo-dimers, -hexamers, and -octamers are non-physiological. Interestingly, no hexameric CpE pre-pore assembly was predicted using this method. Yet, one predicted tetramer recapitulated a potential oligomeric pre-pore and had hallmarks of a physiological form, including claudin orientation and proper Asp48, a pore-forming helix, and nCpE positioning. As this tetramer was generated from a CpE_Tryp_ monomer, we then show how the tetramer could be formed from two CpE_Tryp_ Interface 1 dimers via a minute translation and −90 or 270° rotation. These findings lead us to propose that the CpE_Tryp_ Interface 1 homo-dimer could be a building block of larger oligomeric assemblies and that the cytotoxic β-pore may be a constituent of two. If true, the hexamer proposed by others would qualify, but from these analyses, a tetramer would qualify as well. Indeed, the molecular weight estimates found from native-PAGE or SEC of CpE β-pores exhibit large ranges, from 155 to 660 kDa, depending on the method and cell line [7]. The low resolution and influence of the shape of the complex and detergents/lipids on these analyses—and the CpE construct being used—explains this broad range. Originally, the CpE cytotoxic complex was shown to be 155–210 kDa [18,19]. A CpE_Tryp_ (32 kDa) tetramer bound to four claudin-4 s (22 kDa) would be 216 kDa. The CpE_Tryp_ tetramer may, therefore, have biological significance that manifests through simple rearrangements of two Interface 1 homo-dimers. Higher order oligomers could be formed from the incorporation of other Interface 1 dimers into hexamers (324 kDa) or even decamers that would amass to 540 kDa, which would all fit within the range of experimentally determined masses. More quantitative biophysical and biochemical characterization would illuminate the true mass and stoichiometry of the claudin-bound CpE β-pore, which can be further validated by structure determination of membrane-penetrating β-pore.

Here, we quantify the effect of trypsin treatment on CpE using biophysical methods (MS and SEC-MALS-SAXS) and determine the structural basis of trypsinization on CpE homo-oligomerization. We show that the removal of the N-terminal 25 residues of CpE by trypsin, which is known from crystallography and demonstrated here with SAXS to be disordered, results in a 4 kDa smaller and more compact CpE with decreased dynamics (entropy). We further show that removal of the first 25 residues exposes previously unexposed surfaces within the linker region between nCpE and cCpE and that this region induces CpE/CpE oligomerization primarily through newfound cCpE/nCpE interfaces. We propose that the mechanism of functional activation of CpE by trypsin, which improves three-fold over untreated CpE, likely stems from a combination of entropy decrease and exposure of new oligomeric surfaces through trypsin’s removal of CpE N-terminal residues. Trypsin thus reduces the thermodynamic energy barrier to oligomerization, accelerating the rate and potentially the magnitude of oligomers that CpE can form.

## 4. Conclusions

This work demonstrates the complementarity between solution-based SAXS and X-ray crystallography in determining structures of CpE and provides new insights into CpEs’ pathophysiological mechanism of action. In combination with biophysical analyses, we uncover trypsin’s role in modifying CpE structure and its resulting effect on CpE homo-oligomerization. These findings begin to elucidate the process of CpE oligomerization on its path toward the formation of cytotoxic β-pores—the effects of which irreversibly alter gut homeostasis and lead to serious and prevalent illnesses in domesticated animals and humans. The insights provided here can be applied to target dimer interfaces with therapeutic molecules that trap or inhibit nascent CpE oligomerization as a means to prevent the building of cytotoxic β-pores, which ultimately could lead to treatments for gastrointestinal diseases induced by CpE.

## 5. Materials and Methods

### 5.1. Protein Expression and Purification

CpE and cCpE were expressed and purified as previously described [20]. Briefly, plasmid pFastBac1 (Life Technologies) encoding full-length wild-type CpE with a native N-terminus was expressed in *Trichoplusia ni* Tn5 cells (Expression systems) with a C-terminal decahistidine tag preceded by a thrombin cleavage site. For cCpE, residues 194–319 were cloned downstream of an N-terminal decahistidine followed by enhanced green fluorescent protein, then a thrombin cleavage site that preceded cCpE, which was subcloned into pFastBac1 and expressed in *Spodoptera frugiperda* Sf9 cells (Expression systems). Both proteins were purified via immobilized metal-affinity chromatography (IMAC) with Ni-NTA resin with resin capture, wash, and treatment with thrombin to release proteins. Cleaved proteins were analyzed for purity using SDS-PAGE and analytical size-exclusion chromatography (SEC), then used for SAXS and/or crystallization experiments.

Post-IMAC pure CpE was trypsin digested using immobilized trypsin (ProteoChem) using a 1:5 trypsin/CpE ratio (mass/mass) in 50 mM Tris pH 8.0, 200 mM NaCl, and 5% glycerol overnight at 4 °C. The immobilized trypsin resin was captured, and flow through containing trypsinized CpE (CpE_Tryp_) was collected and then analyzed by SDS-PAGE and analytical SEC for purity.

For crystallization, both post-IMAC pure cCpE and CpE_Tryp_ were run on a Superdex 200 Increase column. For CpE_Tryp_, the mobile phase was 20 mM MES pH 6.5, 100 mM NaCl, and 4% glycerol, while for cCpE, it was 10 mM HEPES pH 7.4, 125 mM NaCl, and 3% glycerol. CpE_Tryp_ was concentrated to 8 mg/mL, while cCpE was concentrated to 6–10 mg/mL and used for crystallization.

### 5.2. Mass Spectrometry Analysis

Post-IMAC pure CpE and CpE_Tryp_ were excised from an SDS-PAGE gel, then washed with water, reduced with 10 mM dithiothreitol, and alkylated with 10 mM iodoacetamide. The gels were washed further in ammonium bicarbonate/acetonitrile to remove SDS and Coomassie brilliant blue stain. Gel fragments were incubated with trypsin, and digestion was carried out overnight at 37 °C. Peptides were extracted from the gel pieces and dried down in a Speed-Vac. The digests were re-dissolved in 5% acetonitrile and 0.5% formic acid. Analysis was carried out using a 1 h gradient on a 0.075 mm × 250 mm C18 Waters CSH column feeding into an Orbitrap Eclipse mass spectrometer run in OT-OT-HCD mode. All MS/MS samples were analyzed using Mascot version 2.7.0 (Matrix Science, London, UK). Mascot was set up to search the cRAP_20150130.fasta (125 entries); uniprot-refprot_Clostridium_perfringens_UP000000818_ 20230629.fasta (2721 entries); and Custom6_20230629 database (1 entry) for semi tryptic peptides. Mascot was searched with a fragment ion mass tolerance of 0.060 Da and a parent ion tolerance of 10.0 parts per million. Deamidation of asparagine and glutamine, oxidation of methionine, and carbamidomethyl of cysteine were specified in Mascot as variable modifications. Scaffold 5.2.2 (Proteome Software Inc., Portland, OR, USA) was used to validate MS/MS-based peptide and protein identifications. Peptide identifications were accepted if they could be established at greater than 95.0% probability by the Peptide Prophet algorithm [21] with Scaffold delta-mass correction. Protein identifications were accepted if they could be established at greater than 99.9% probability and contained at least two identified peptides. Protein probabilities were assigned by the Protein Prophet algorithm [22]. Proteins that contained similar peptides and could not be differentiated based on MS/MS analysis alone were grouped to satisfy the principles of parsimony. Proteins sharing significant peptide evidence were grouped into clusters.

### 5.3. SEC-MALS-SAXS Data Collection and Analysis

SAXS was performed at BioCAT (beamline 18ID at the Advanced Photon Source, Chicago, IL, USA) with in-line SEC to separate the sample from aggregates and other contaminants, thus ensuring optimal sample quality and multiangle light scattering (MALS), dynamic light scattering (DLS) and refractive index measurement (RI) for additional biophysical characterization. The samples were loaded on a Superdex 200 Increase 10/300 GL column (Cytiva) run by a 1260 Infinity II HPLC (Agilent Technologies) at 0.6 mL/min. The flow passed through (in order) the Agilent UV detector, a MALS detector and DLS detector (DAWN Helios II, Wyatt Technologies), and an RI detector (Optilab T-rEX, Wyatt). The flow then went through the SAXS flow cell. The flow cell consists of a 1.0 mm ID quartz capillary with ~20 μm walls. A coflowing buffer sheath is used to separate samples from the capillary walls, helping prevent radiation damage [23]. Scattering intensity was recorded using an Eiger2 XE 9M (Dectris) detector, which was placed 3.6 m from the sample, giving us access to a q-range of 0.0045 Å^−1^ to 0.35 Å^−1^. During elution, 0.5 s exposures were acquired every 2 s, and data were reduced using BioXTAS RAW 2.1.4 [24]. Buffer blanks were created by averaging regions flanking the elution peak (For CpE, frames 572–661 and 1295–1380 and, for CpE_Tryp_, frames 456–531 and 1320–1355 were used for buffer subtraction) and subtracted from exposures selected from the elution peak (For CpE, frames 812–818 and, for CpE_Tryp_, frames 836–846 were used) to create the I(q) vs. q curves used for subsequent analyses. The resulting subtracted scattering profile was analyzed to obtain Guinier fit and molecular weight estimation using BioXTAS RAW and p(r) function using GNOM [24]. Molecular weights and hydrodynamic radii were calculated from the MALS and DLS data, respectively, using the ASTRA 7 software (Wyatt). Data were visualized and plotted with GraphPad Prism version 9.5.1. Reconstruction of the SAXS envelope from the 2D scattering curve was performed with GASBOR [25]. SAXS-based model was aligned with the crystal structure using CIFSUP, and the model-to-map fit was assessed with FoXS [26]. Outputs were analyzed further and visualized using PyMol (The PyMOL Molecular Graphics System, version 2.4, Schrödinger LLC). Table S2 summarizes information on data collection and processing. To generate the missing 1–37 residues in the crystal structure of CpE_Tryp_, Ensemble Optimization Method (EOM) available as webserver was used [27].

### 5.4. Crystallization and Structure Determination

CpE_Tryp_ crystals grew from a mother liquor containing 100 mM sodium phosphate monobasic monohydrate, 100 mM potassium phosphate monobasic, 100 mM MES monohydrate pH 6.5, and 2.0 M sodium chloride using 300 µL in well and 1:1 µL protein/cocktail. Crystals appeared after seven days at 4 °C and were then harvested after cryoprotecting using 50% glycerol and flash freezing in liquid nitrogen. Diffraction data from a single crystal were collected at Advanced Photon Source GM/CA Beamline 23ID-B. Data were initially processed in space group P 4_1_ 2_1_ 2 using XDS [28]. Matthew’s coefficient calculations hinted that 14–18 copies of CpE_Tryp_ were in the asymmetric unit. However, attempts at phasing with molecular replacement using Phaser [29] failed to produce a model when searching for 14–18 copies of PDB ID 3ZIW or 2YHK. Using P 4_1_ 2_1_ 2 processed data, MoRDa [30] found several correct solutions, but all were obtained in space group P 4_3_ 2 2 and contained eight copies of CpE_Tryp_. Subsequently, diffraction images were re-processed in P 4_3_ 2 2 with XDS. Using X-ray intensities from XDS input into Phaser, we searched for eight copies of CpE using an output .pdb file from MoRDa. Phaser successfully found eight copies (74% solvent content) with LLG and TFZ scores of 22,654 and 54.2 in P 4_3_ 2 2. The Phaser output .pdb was auto-built using phenix_autobuild and ARP/wARP and then iteratively refined using phenix_refine [31,32,33]. The final R/R_free_ obtained post-refinement was 22/25%. Table S4 contains all data and statistics from crystallographic analyses.

The cCpE crystals grew from a mother liquor composed of 100 mM sodium acetate pH 4.5, 200 mM sodium chloride, and ammonium sulfate. Two crystals that resulted in structures were grown from two different wells that varied as follows—cocktail as above with 1.3 M ammonium sulfate with 167 µL in well and 1:1 µL in the drop and cocktail as above with 1.0 M ammonium sulfate with 200 µL in well and 1:1 µL in the drop. Crystals in each well exhibited distinct morphologies and grew from 3 to 14 days at 19 °C. Each of the two drops was spiked with 0.5 µL of 50% glycerol to cryoprotect crystals just before flash freezing in liquid nitrogen. Data were collected at Beamline 8.3.1 at the Advanced Light Source at Berkeley National Lab using a Pilatus detector and processed in XDS with space group verification in Pointless. The space group for the crystal grown in 1.3 M ammonium sulfate was determined to be P 4_1_ 2_1_ 2, while for the crystal grown in 1.0 M ammonium sulfate, it was P 2_1_ 2_1_ 2_1_. Structures were determined by molecular replacement using only the cCpE domain (residues 204–319) of PDB ID: 3AM2 as a search model [13]. Multiple cycles of refinement were performed using phenix.refine waters were located, and glycerol and acetate that were part of the crystallization cocktails were identified during the process of refinement. R_work_/R_free_ for the two cCpE structures were fully refined to 16/20% and 19/22% (Table S4).

### 5.5. Computational Modeling of Claudin-Bound CpE Oligomers

We superposed cCpE from PDB ID: 6OV2 onto the cCpE domain of all six dimer interfaces of CpE_Tryp_ using Coot [34]. This placed claudin, which is bound to cCpE in 6OV2, in its experimentally determined orientation. We then used PDBePISA to search the PDB for other interfaces that resembled Interface 1 [15]. PDBePISA searched 170,428 structures and 4,348,532 interfaces and found 7 similar interfaces. We examined these by superposing cCpE and claudin from 6OV2 as before to estimate the physiological relevance of PDB IDs 2YHJ, 2XH6, 3AM2, 3ZIW, 3ZIX, 3ZJ3, and 4P5H. Interfaces were not considered physiologically relevant if the claudins were not organized perpendicular to the membrane plane. Some of these findings appear in Figure S4.

Chain A from CpE_Tryp_ was used to predict the homo-oligomerization of CpE using GalaxyHomomer [35]. For this, we searched independently for dimers, tetramers, hexamers, and octamers. Five models for each oligomer queried (20 total) were output and analyzed by superposing cCpE from 6OV2 onto the cCpE domain of all CpE chains. Because only one tetramer appeared physiologically relevant, we modeled the conformational change that two Interface 1 dimers would take to form this tetramer by generating structural morphs with PyMol (The PyMOL Molecular Graphics System, version 2.4, Schrödinger LLC). These results appear in Movie S1 and S2.

## Figures and Tables

**Table 1:** Interaction interfaces observed in the crystal structure of CpE_Tryp_.

	Structure 1	N_res_	Surface, Å	Structure 2	N_res_	Surface, Å	Average Interface Area, Å	Average ΔG kcal/mol	Average N_HB_	Average N_SB_
Interface 1	B, H	18, 17	13,943, 14,208	A, G	18, 17	13,986, 13,941	573.1	−3.5	5	0
Interface 2	D, C, G	21, 21, 19	13,804, 13,843, 13,941	B, A, E	18, 18, 15	13,943, 13,986, 13,916	510.2	−2.6	6	1
Interface 3	E, H	11, 11	13,916, 14,208	B, C	13, 10	13,943, 13,843	361.1	0.4	5	0
Interface 4	E	14	13,916	C	14	13,843	326.2	−4.2	2	0
Interface 5	G, E	17, 9	13,941, 13,916	C, A	11, 17	13,843, 13,986	358.9	−2.8	4	0
Interface 6	F	12	13,851	A	18	13,986	231.2	−2.3	0	0

## Data Availability

The coordinate and structure factor files for the crystal structures mentioned in this research paper are deposited in RCSB PDB. The PDB IDs are as follows: 8U5D for cCpE in space group P 4_1_ 2_1_ 2; 8U5E for cCpE in space group P 2_1_ 2_1_ 2_1_; and 8U5F for CpE_Tryp_. Data from SAXS were deposited in the SASBDB. The SASBDB IDs are as follows: SASDSH9 for CpE and SASDSJ9 for CpE_Tryp_.

## Supplementary Materials

The following supporting information can be downloaded at https://www.mdpi.com/article/10.3390/toxins15110637/s1. Figure S1: Additional SEC-MALS SAXS data; Figure S2: Asymmetric unit content and protein packing of CpE crystal structures; Figure S3: Structural comparison of CpE monomers from various crystal structures; Figure S4: Models of previously determined CpE structures bound to Claudin; Table S1: List of unique peptides identified by mass spectrometry for CpE and CpE_Tryp_: Table S2: SAXS data collection statistics; Table S3: R_g_ and D_max_ of EOM generated models of CpE; Table S4: Crystallographic data collection and refinement statistics. Movie S1: Structural model of CpE_Tryp_ Interface 1 dimer transition to tetramer (top view). Movie S2: Structural model of CpE_Tryp_ Interface 1 dimer transition to tetramer (side view). Refs. [36,37,38,39,40] are cited in the Supplementary Materials.
