# Supplementary material for: Structural Basis of Clostridium perfringens Enterotoxin Activation and Oligomerization by Trypsin
Source: Toxins (Basel). 2023 Oct 31;15(11):637. doi: 10.3390/toxins15110637 (PMC10674488; doi:10.3390/toxins15110637)
Supplement: Supplementary file 1 [file toxins-15-00637-s001.zip › toxins-2641006-supplementary.pdf]

*Supplementary Information for*

# Structural Basis of *Clostridium perfringens* Enterotoxin Activation and Oligomerization by Trypsin

**Chinemerem P. Ogbu[1,2], Srajan Kapoor[1,2], and Alex J. Vecchio[1,*]**

1 Department of Structural Biology, University at Buffalo, The State University of New York, Buffalo, New York 14203, USA; ORCID 0000-0001-5663-7148, cpogbu@buffalo.edu (C.P.O); ORCID 0000-0003-1347-550X kapoorsrajan@gmail.com (S.K.); ORCID 0000-0002-4222-7874, vecchioa@buffalo.edu (A.J.V.)
2 Equal Contribution
* Correspondence: vecchioa@buffalo.edu

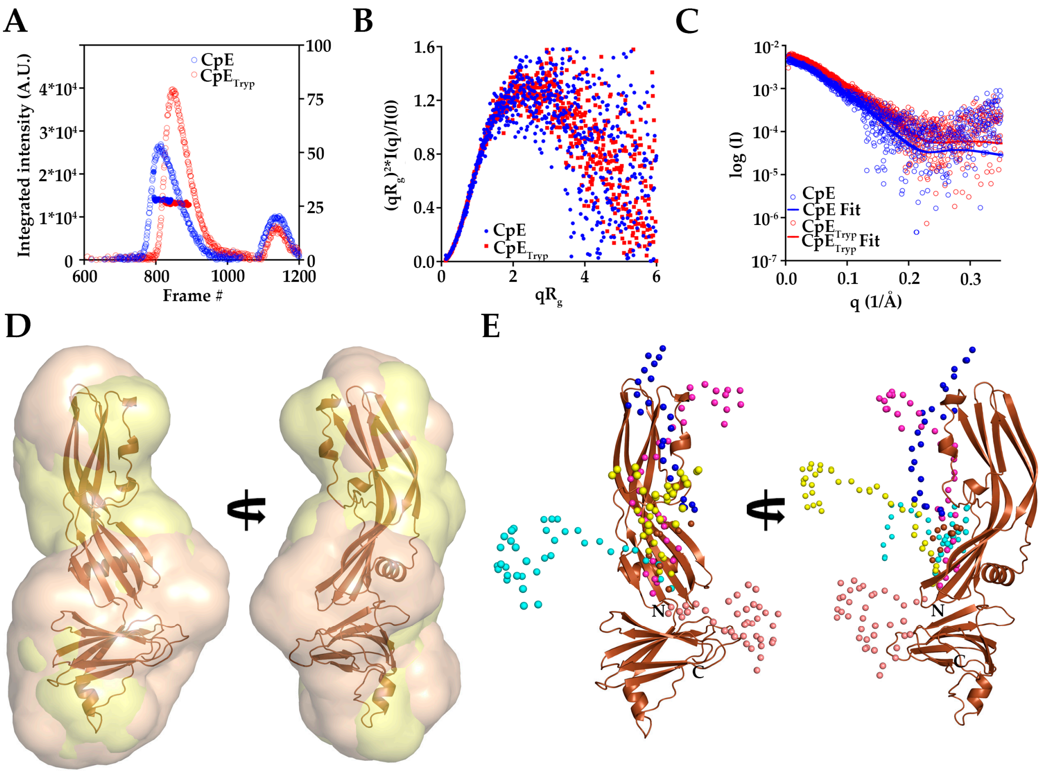


**Figure S1: Additional SEC-MALS SAXS results.** (A) Subtracted series plots showing integrated intensity *I*(0) (hollow circles) and Rg (filled circles) versus the frame number for the SEC-MALS-SAXS run for CpE (blue) and $CpE_{Tryp}$ (red). (B) dimensionless Kratky plot of CpE (Blue) and $CpE_{Tryp}$ (Red) shows that the protein is properly folded. (C) FoXS based fitting of a theoretical scattering profile of CpE (PDB ID: 2XH6) to the experimental SAXS profile of CpE (Blue) and $CpE_{Tryp}$ (Red). (D) Overlay of SAXS envelop of CpE (Wheat) and $CpE_{Tryp}$ (Yellow) and 2XH6. (E) EOM generated five different models of CpE resides 1-37 that were missing from the crystal structure [1,2]. Out of the five EOM generated models, one (blue spheres) showed the $D_{max}$ of ~103 Å and $R_g$ of ~ 27 Å other models therefore this model was selected for further analysis. The $D_{max}$ and Rg for five different EOM generated models are given in Table S4.

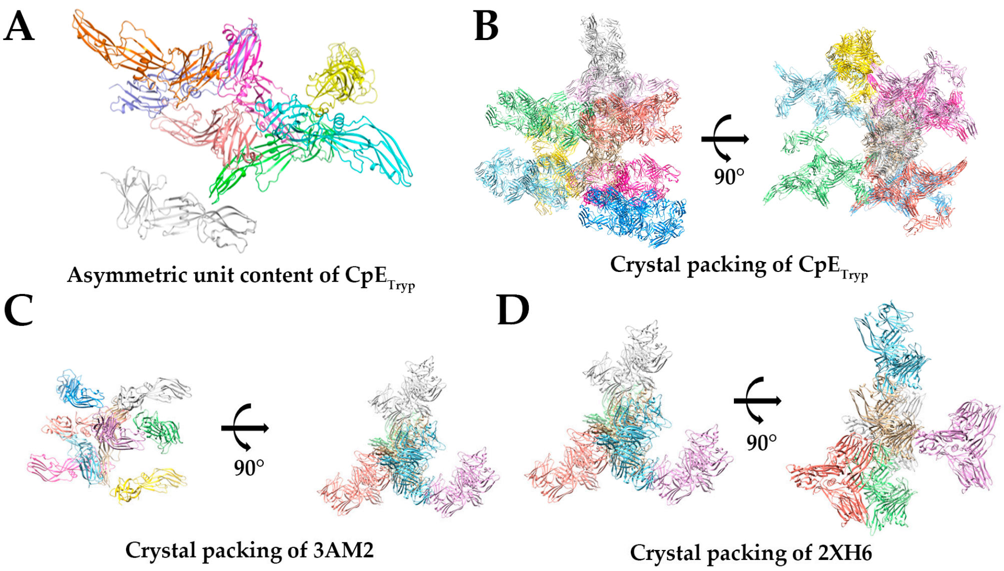


**Figure S2: Asymmetric Unit Content and Protein Packing of CpE Crystal Structures.** (A) Asymmetric unit of CpE$_{Tryp}$ with eight molecules each coloured independently. Crystal packing of (B) CpE$_{Tryp}$, (C) PDB ID: 3AM2, and (D) PDB ID: 2XH6. Each monomer is coloured uniquely

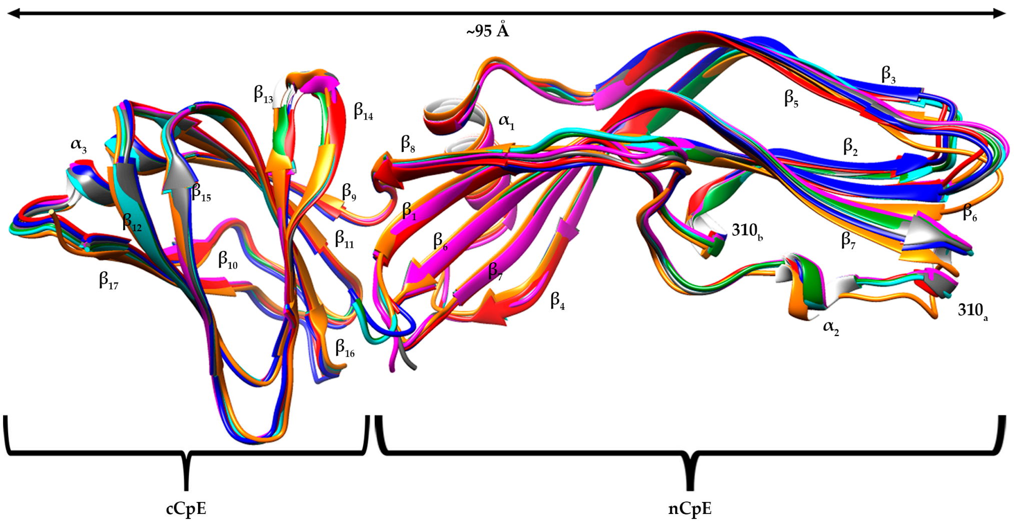


**Figure S3: Structure Comparison of CpE Monomers from Various Crystal Structures.** Structural overlay of $CpE_{Tryp}$ chain A (cyan) with those from PDB ID: 2XH6 (orange), 6YHJ (green), 3AM2 (red), 3ZIW (blue), and 4P5H (magenta). CpE is shown as a cartoon.

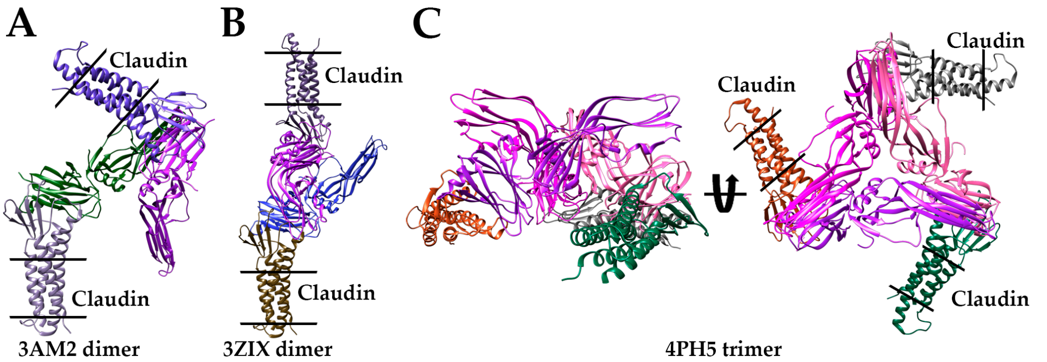


**Figure S4: Models of Previously Determined CpE Structures Bound to Claudins.** Superpositioning of claudins onto the dimer from PDB ID: 3AM2 (left), 3ZIX (middle), and trimer from 4PH5 (right) shows that claudins are not primed in physiologically relevant poses.

**Table S1. List of unique peptides identified by mass spectrometry for CpE and CpE$_{Tryp}$**

| Unique peptide number | Unique peptides | Peptide count CpE | Peptide count CpE$_{Tryp}$ |
|---|---|---|---|
| 1 | MLSNNLNPM | 2 | 0 |
| 2 | MLSNNLNPMVFEN | 4 | 0 |
| 3 | MLSNNLNPMVFENAK | 292 | 22 |
| 4 | LSNNLNPMVFENAK | 8 | 0 |
| 5 | SNNLNPMVFENAK | 15 | 0 |
| 6 | NNLNPMVFENAK | 72 | 2 |
| 7 | NLNPMVFENAK | 25 | 1 |
| 8 | LNPMVFENAK | 11 | 0 |
| 9 | NPMVFENAK | 4 | 0 |
| 10 | PMVFENAK | 22 | 0 |
| 11 | ENAKEVFLISEDLK | 1 | 0 |
| 12 | AKEVFLISEDLK | 4 | 0 |
| 13 | KEVFLISEDLK | 2 | 0 |
| 14 | EVFLISEDLK | 137 | 9 |
| 15 | EVFLISEDLKTPINITNSNSNLSDGLYVIDK | 1 | 0 |
| 16 | VFLISEDLK | 2 | 0 |
| 17 | FLISEDLK | 7 | 0 |
| 18 | LISEDLKTPINITNSNSNLSDGLYVIDK | 1 | 0 |
| 19 | LKTPINITNSNSNLSDGLYVIDK | 1 | 0 |
| 20 | KTPINITNSNSNLSDGLYVIDK | 0 | 2 |
| 21 | TPINITNSN | 7 | 6 |
| 22 | TPINITNSNS | 1 | 1 |
| 23 | TPINITNSNSN | 8 | 7 |
| 24 | TPINITNSNSNL | 2 | 2 |
| 25 | TPINITNSNSNLS | 1 | 1 |
| 26 | TPINITNSNSNLSD | 4 | 3 |
| 27 | TPINITNSNSNLSDG | 1 | 1 |
| 28 | TPINITNSNSNLSDGL | 1 | 2 |
| 29 | TPINITNSNSNLSDGLY | 0 | 1 |
| 30 | TPINITNSNSNLSDGLYVIDK | 121 | 133 |
| 31 | TPINITNSNSNLSDGLYVIDKGD | 2 | 2 |
| 32 | TPINITNSNSNLSDGLYVIDKGDG | 1 | 3 |
| 33 | PINITNSNSNLSDGLYVIDK | 3 | 4 |
| 34 | INITNSNSNLSDGLYVIDK | 4 | 6 |
| 35 | NITNSNSNLSDGLYVIDK | 8 | 6 |
| 36 | ITNSNSNLSDGLYVIDK | 9 | 11 |
| 37 | TNSNSNLSDGLYVIDK | 2 | 2 |
| 38 | NSNSNLSDGLYVIDK | 7 | 10 |
| 39 | SNSNLSDGLYVIDK | 15 | 18 |
| 40 | NSNLSDGLYVIDK | 3 | 4 |
| 41 | SNLSDGLYVIDK | 5 | 9 |
| 42 | NLSDGLYVIDK | 1 | 2 |
| 43 | LSDGLYVIDK | 8 | 7 |
| 44 | SDGLYVIDK | 1 | 2 |
| 45 | DGLYVIDK | 1 | 1 |
| 46 | GDGWILGEPS | 1 | 1 |
| 47 | GDGWILGEPSVVSSQILNPNETGTFSQSLTK | 5 | 1 |
| 48 | GEPSVVSSQILNPNETGTFSQSLTK | 4 | 4 |
| 49 | EPSVVSSQILNPNETGTFSQSLTK | 1 | 2 |
| 50 | PSVVSSQILNPNETGTFSQSLTK | 2 | 1 |
| 51 | SVVSSQILNPNETGTFSQSLTK | 1 | 1 |
| 52 | VVSSQILNPNETGTFSQSLTK | 7 | 9 |
| 53 | SSQILNPNETGTFSQSLTK | 0 | 2 |
| 54 | SQILNPNETGTFSQSLTK | 3 | 4 |
| 55 | QILNPNETGTFSQSLTK | 4 | 4 |
| 56 | ILNPNETGTFSQSLTK | 8 | 9 |
| 57 | NPNETGTFSQSLTK | 2 | 3 |
| 58 | PNETGTFSQSLTK | 8 | 7 |
| 59 | NETGTFSQSLTK | 2 | 2 |
| 60 | ETGTFSQSLTK | 5 | 4 |
| 61 | TGTFSQSLTK | 2 | 1 |
| 62 | GTFSQSLTK | 2 | 2 |
| 63 | TFSQSLTK | 3 | 3 |
| 64 | SKEVSINVN | 1 | 1 |
| 65 | FGITIGEQNTIER | 1 | 1 |

| | | | |
|---|---|---|---|
| 66 | ITIGEQNTIER | 1 | 2 |
| 67 | IGEQNTIER | 1 | 1 |
| 68 | SVSTTAGPN | 1 | 1 |
| 69 | SVSTTAGPNEY | 1 | 1 |
| 70 | SVSTTAGPNEYVYYK | 153 | 150 |
| 71 | SVSTTAGPNEYVYYKVYATYR | 3 | 3 |
| 72 | SVSTTAGPNEYVYYKVYATYRK | 6 | 7 |
| 73 | SVSTTAGPNEYVYYKVYATYRKYQAIR | 3 | 5 |
| 74 | VSTTAGPNEYVYYK | 2 | 2 |
| 75 | STTAGPNEYVYYK | 2 | 5 |
| 76 | TTAGPNEYVYYK | 2 | 2 |
| 77 | TAGPNEYVYYK | 3 | 3 |
| 78 | AGPNEYVYYK | 4 | 5 |
| 79 | GPNEYVYYK | 1 | 1 |
| 80 | PNEYVYYK | 3 | 2 |
| 81 | RISHGNISDDGSIYK | 0 | 1 |
| 82 | ISHGNISD | 1 | 1 |
| 83 | ISHGNISDD | 1 | 2 |
| 84 | ISHGNISDDG | 1 | 1 |
| 85 | ISHGNISDDGS | 1 | 1 |
| 86 | ISHGNISDDGSIYK | 268 | 251 |
| 87 | ISHGNISDDGSIYKL | 1 | 1 |
| 88 | ISHGNISDDGSIYKLTGIWLSK | 2 | 5 |
| 89 | SHGNISDDGSIYK | 4 | 3 |
| 90 | HGNISDDGSIYK | 6 | 7 |
| 91 | GNISDDGSIYK | 4 | 2 |
| 92 | NISDDGSIYK | 1 | 1 |
| 93 | ISDDGSIYK | 6 | 5 |
| 94 | SDDGSIYK | 3 | 2 |
| 95 | KLTGIWLSK | 1 | 1 |
| 96 | LTGIWLSK | 85 | 81 |
| 97 | LTGIWLSKTSADSLGNIDQGSLIETGER | 2 | 2 |
| 98 | SKTSADSLGNIDQGSLIETGER | 1 | 1 |
| 99 | KTSADSLGNIDQGSLIETGER | 1 | 2 |
| 100 | TSADSLGNID | 1 | 1 |
| 101 | TSADSLGNIDQ | 2 | 1 |
| 102 | TSADSLGNIDQG | 1 | 3 |
| 103 | TSADSLGNIDQGS | 2 | 2 |
| 104 | TSADSLGNIDQGSLIE | 1 | 1 |
| 105 | TSADSLGNIDQGSLIET | 1 | 2 |
| 106 | TSADSLGNIDQGSLIETG | 2 | 2 |
| 107 | TSADSLGNIDQGSLIETGE | 2 | 4 |
| 108 | TSADSLGNIDQGSLIETGER | 136 | 160 |
| 109 | TSADSLGNIDQGSLIETGERCVLTVPSTDIEK | 2 | 3 |
| 110 | SADSLGNIDQGSLIETGER | 2 | 3 |
| 111 | ADSLGNIDQGSLIETGER | 0 | 4 |
| 112 | DSLGNIDQGSLIETGER | 2 | 3 |
| 113 | SLGNIDQGSLIETGER | 7 | 5 |
| 114 | LGNIDQGSLIETGER | 4 | 4 |
| 115 | GNIDQGSLIETGER | 3 | 5 |
| 116 | NIDQGSLIETGER | 6 | 7 |
| 117 | IDQGSLIETGER | 3 | 5 |
| 118 | DQGSLIETGER | 0 | 1 |
| 119 | QGSLIETGER | 4 | 4 |
| 120 | GSLIETGER | 2 | 2 |
| 121 | SLIETGER | 2 | 1 |
| 122 | CVLTVPSTDIEK | 22 | 27 |
| 123 | CVLTVPSTDIEKEILDLAAATER | 60 | 50 |
| 124 | VLTVPSTDIEK | 2 | 2 |
| 125 | VLTVPSTDIEKEILDLAAATER | 0 | 2 |
| 126 | LTVPSTDIEK | 1 | 0 |
| 127 | LTVPSTDIEKEILDLAAATER | 3 | 0 |
| 128 | TVPSTDIEK | 5 | 3 |
| 129 | TVPSTDIEKEILDLAAATER | 2 | 2 |
| 130 | PSTDIEKEILDLAAATER | 3 | 2 |
| 131 | IEKEILDLAAATER | 2 | 0 |
| 132 | EKEILDLAAATER | 0 | 1 |
| 133 | KEILDLAAATER | 1 | 0 |

| | | | |
|---|---|---|---|
| 134 | EILDLAAATER | 50 | 68 |
| 135 | ILDLAAATER | 2 | 2 |
| 136 | LDLAAATER | 1 | 3 |
| 137 | DLAAATER | 1 | 1 |
| 138 | LNLTDALN | 0 | 1 |
| 139 | LNLTDALNSN | 3 | 4 |
| 140 | LNLTDALNSNPA | 2 | 2 |
| 141 | LNLTDALNSNPAG | 2 | 3 |
| 142 | LNLTDALNSNPAGN | 3 | 6 |
| 143 | LNLTDALNSNPAGNLYDWR | 84 | 87 |
| 144 | NLTDALNSNPAGNLYDWR | 2 | 4 |
| 145 | LTDALNSNPAGNLYDWR | 4 | 8 |
| 146 | TDALNSNPAGNLYDWR | 2 | 3 |
| 147 | DALNSNPAGNLYDWR | 0 | 2 |
| 148 | ALNSNPAGNLYDWR | 8 | 7 |
| 149 | LNSNPAGNLYDWR | 1 | 2 |
| 150 | NSNPAGNLYDWR | 2 | 3 |
| 151 | SNPAGNLYDWR | 7 | 8 |
| 152 | NPAGNLYDWR | 2 | 2 |
| 153 | PAGNLYDWR | 11 | 13 |
| 154 | AGNLYDWR | 1 | 1 |
| 155 | RSSNSYPWTQK | 1 | 2 |
| 156 | SSNSYPWTQK | 160 | 191 |
| 157 | SSNSYPWTQKLNLHLTITATGQK | 33 | 23 |
| 158 | SSNSYPWTQKLNLHLTITATGQKYR | 17 | 15 |
| 159 | SSNSYPWTQKLNLHLTITATGQKYRILASK | 2 | 1 |
| 160 | SNSYPWTQK | 3 | 6 |
| 161 | NSYPWTQK | 3 | 4 |
| 162 | SYPWTQKLNLHLTITATGQK | 1 | 1 |
| 163 | TQKLNLHLTITATGQK | 0 | 1 |
| 164 | KLNLHLTITATGQK | 1 | 0 |
| 165 | LNLHLTIT | 1 | 1 |
| 166 | LNLHLTITAT | 1 | 1 |
| 167 | LNLHLTITATG | 1 | 1 |
| 168 | LNLHLTITATGQ | 1 | 0 |
| 169 | LNLHLTITATGQK | 132 | 110 |
| 170 | LNLHLTITATGQKYR | 9 | 6 |
| 171 | NLHLTITATGQK | 5 | 3 |
| 172 | LHLTITATGQK | 5 | 6 |
| 173 | HLTITATGQK | 3 | 1 |
| 174 | LTITATGQK | 2 | 2 |
| 175 | TITATGQK | 1 | 1 |
| 176 | YRILASKIVDFNIYSNNFNNLVK | 1 | 0 |
| 177 | IVDFNIYSNNFNN | 0 | 1 |
| 178 | IVDFNIYSNNFNNLVK | 80 | 69 |
| 179 | FNIYSNNFNNLVK | 2 | 3 |
| 180 | FNIYSNNFNNLVKLEQSLGDGVK | 0 | 1 |
| 181 | IYSNNFNNLVK | 5 | 5 |
| 182 | SNNFNNLVK | 6 | 6 |
| 183 | NNFNNLVK | 4 | 2 |
| 184 | KLEQSLGDGVK | 2 | 2 |
| 185 | LEQSLGDGVK | 70 | 63 |
| 186 | LEQSLGDGVKD | 1 | 2 |
| 187 | LEQSLGDGVKDH | 3 | 2 |
| 188 | LEQSLGDGVKDHY | 1 | 1 |
| 189 | LEQSLGDGVKDHYVD | 2 | 0 |
| 190 | LEQSLGDGVKDHYVDI | 0 | 1 |
| 191 | LEQSLGDGVKDHYVDISLDAG | 1 | 0 |
| 192 | LEQSLGDGVKDHYVDISLDAGQY | 1 | 1 |
| 193 | LEQSLGDGVKDHYVDISLDAGQYVLVMK | 61 | 49 |
| 194 | EQSLGDGVK | 1 | 1 |
| 195 | QSLGDGVK | 3 | 2 |
| 196 | SLGDGVKDHYVDISLDAGQYVLVMK | 3 | 2 |
| 197 | LGDGVKDHYVDISLDAGQYVLVMK | 1 | 1 |
| 198 | DGVKDHYVDISLDAGQYVLVMK | 4 | 2 |
| 199 | GVKDHYVDISLDAGQYVLVMK | 2 | 2 |
| 200 | VKDHYVDISLDAGQYVLVMK | 0 | 1 |
| 201 | DHYVDISL | 0 | 1 |

| | | | |
|---|---|---|---|
| 202 | DHYVDISLD | 1 | 1 |
| 203 | DHYVDISLDAG | 1 | 1 |
| 204 | DHYVDISLDAGQY | 2 | 1 |
| 205 | DHYVDISLDAGQYVLVMK | 58 | 38 |
| 206 | DHYVDISLDAGQYVLVMKA | 0 | 1 |
| 207 | HYVDISLDAGQYVLVMK | 0 | 1 |
| 208 | ISLDAGQYVLVMK | 2 | 1 |
| 209 | AGQYVLVMK | 2 | 2 |
| 210 | GQYVLVMK | 1 | 1 |
| 211 | KANSSYSGNYPYSILFQK | 0 | 1 |
| 212 | ANSSYSGNYPY | 1 | 0 |
| 213 | ANSSYSGNYPYSILFQK | 89 | 88 |
| 214 | SSYSGNYPYSILFQK | 11 | 16 |
| 215 | YSGNYPYSILFQK | 0 | 2 |
| 216 | SGNYPYSILFQK | 4 | 3 |
| 217 | GNYPYSILFQK | 4 | 3 |
| 218 | NYPYSILFQK | 1 | 1 |
| 219 | YPYSILFQK | 7 | 9 |
| 220 | PYSILFQK | 3 | 4 |

**Table S2. SAXS Data Collection Statistics.**

| **Data collection parameters** | | |
|---|---|---|
| **Instrument** | BioCAT facility at the Advanced Photon Source beamline 18ID with Pilatus3 X 1M (Dectris) detector | |
| Experiment type | SEC-MALS-SAXS | |
| Column | Superdex 200 10/300 Increase (0.6 mL/min) | |
| Wavelength (Å) | 1.033 | |
| Detector distance (m) | 3.628 | |
| q-measurement range ($Å^{-1}$) | 0.0045 – 0.35 | |
| Exposure time (s) | 0.5 | |
| Exposure period (s) | 2.0 | |
| Sample temperature (°C) | 22 | |
| **Sample details** | | |
| Proteins | CpE | $CpE_{Tryp}$ |
| Solvent composition | 10 mM HEPES pH 7.4, 100 mM NaCl, 2% glycerol | |
| **Analysis statistics** | | |
| ***Guinier analysis*** | | |
| $I(0)$ ($cm^{-1}$) | 0.00431 ± 0.000037 | 0.0059 ± 0.000029 |
| $R_g$ (Å) | 27.46 ± 0.42 | 26.02 ± 0.23 |
| $qR_g$ range | 0.28 – 1.29 | 0.27 – 1.29 |
| Coefficient of correlation, $R^2$ | 0.90 | 0.96 |
| ***P(r) analysis*** | | |
| $I(0)$ ($cm^{-1}$) | 0.00437 ± 0.000041 | 0.00598 ± 0.000030 |
| $R_g$ (Å) | 28.92 ± 0.39 | 27.4 ± 0.22 |
| Dmax (Å) | 102 | 95 |
| **Scattering particle size** | | |
| ***Volume estimates*** | | |
| Porod volume ($Å^{-3}$) | 50200 | 35500 |
| ***MW Estimates (kDa)*** | | |
| Vc MW [3] | 34.4 | 26.7 |
| Vp MW [4] | 41.7 | 29.5 |
| Bayesian MW (% confidence) [5] | 35.8 – 39.8 (95.3%) | 27.9 – 31.3 (90.6%) |
| Shapes and size MW [6] | 37.8 | 30.0 |
| SASBDB ID | SASDH9 | SASDSJ9 |

**Table S3: $R_g$ and $D_{max}$ of EOM generated models of CpE**

| **Model** | **$R_g$ (Å)** | **$D_{max}$ (Å)** |
|---|---|---|
| 1 (Salmon spheres) | 27.00 | 96.90 |
| 2 (Yellow spheres) | 29.40 | 96.90 |
| 3 (Magenta spheres) | 27.10 | 96.90 |
| **4 (Blue spheres)** | **27.20** | **103.30** |
| 5 (Cyan spheres) | 29.00 | 96.90 |

**Table S4. Crystallographic data collection and refinement statistics**

| | cCpE Dimer | cCpE Tetramer | $CpE_{Tryp}$ |
|---|---|---|---|
| Beamline | Beamline ALS 8.3.1 | Beamline ALS 8.3.1 | Beamline APS 23ID-B |
| Wavelength (Å) | 1.11583 | 1.11583 | 1.033167 |
| Space group | P 41 21 2 | P 21 21 21 | P 43 2 2 |
| Cell dimensions | | | |
| a, b, c (Å) | 65.12, 65.12, 130.77 | 65.63, 64, 136.98 | 200.33, 200.33, 254.78 |
| α, β, ɣ (°) | 90, 90, 90 | 90, 90, 90 | 90, 90, 90 |
| Resolution range (Å)[a] | 19.7 - 1.6 (1.657 - 1.6) | 57.18 - 1.4 (1.45 - 1.4) | 66.78 - 2.32 (2.403 - 2.32) |
| Total reflections | 212098 (15496) | 409546 (8980) | 3129407 (242167) |
| Unique reflections | 70024 (5197) | 186626 (5803) | 222147 (16289) |
| Multiplicity | 1.73 (2.98) | 2.19 (1.55) | 14.09 (14.89) |
| Completeness (%)[b] | 99.52 (99.92) | 88.96 (41.76) | 99.62 (97.37) |
| Mean I/SigmaI | 10.8 (0.42) | 9.55 (0.29) | 7.17 (0.39) |
| CC1/2 | 99.9 (12.7) | 99.9 (12.5) | 99.7 (11.8) |
| Wilson B factor | 28.04 | 20.32 | 64.65 |
| $R_{work}$/ $R_{free}$ | 0.196/0.222 | 0.161/0.204 | 0.224/0.248 |
| RMSD | | | |
| Bond length (Å) | 0.014 | 0.005 | 0.004 |
| Bond angle (°) | 1.47 | 1.06 | 0.95 |
| Ramachandran (%) | | | |
| Favored | 98.33 | 98.59 | 97.15 |
| Allowed | 1.67 | 1.41 | 2.76 |
| Outliers | 0.00 | 0.00 | 0.09 |
| Rotamer outliers | 0.00 | 0.00 | 0.40 |
| Clash score | 11.67 | 9.76 | 14.22 |
| Average B factors | 39.63 | 28.74 | 76.22 |
| Molprobity score[c] | 1.57 | 1.52 | 1.81 |
| PDB ID[d] | 8U5D | 8U5E | 8U5F |

[a]Values in parenthesis are for highest resolution shell.
[b]Percentage of correlation between intensities from random half-datasets. Correlation significant at the 0.1% level (CC1/2), as calculated by XDS [7].
[c]As determined by http://molprobity.biochem.duke.edu/.
[d]Deposition code in the Protein Data Bank.

**Movie S1: Structural Model of CpE$_{Tryp}$ Interface 1 Dimer Transition to Tetramer (Top View).** Structural morph depicting the conformational change from two CpE$_{Tryp}$ Interface 1 dimers to form the predicted tetramer.

**Movie S2: Structural Model of CpE$_{Tryp}$ Interface 1 Dimer Transition to Tetramer (Side View).** Structural morph depicting the conformational change from two CpE$_{Tryp}$ Interface 1 dimers to form the predicted tetramer.

**References**

1. Tria, G.; Mertens, H.D.T.; Kachala, M.; Svergun, D.I. Advanced Ensemble Modelling of Flexible Macromolecules Using X-Ray Solution Scattering. *IUCrJ* **2015**, *2*, 207–217, doi:10.1107/S205225251500202X.
2. Bernadó, P.; Mylonas, E.; Petoukhov, M.V.; Blackledge, M.; Svergun, D.I. Structural Characterization of Flexible Proteins Using Small-Angle X-Ray Scattering. *J. Am. Chem. Soc.* **2007**, *129*, 5656–5664, doi:10.1021/ja069124n.
3. Rambo, R.P.; Tainer, J.A. Accurate Assessment of Mass, Models and Resolution by Small-Angle Scattering. *Nature* **2013**, *496*, 477–481, doi:10.1038/nature12070.
4. Piiadov, V.; Ares de Araújo, E.; Oliveira Neto, M.; Craievich, A.F.; Polikarpov, I. SAXSMoW 2.0: Online Calculator of the Molecular Weight of Proteins in Dilute Solution from Experimental SAXS Data Measured on a Relative Scale. *Protein Sci. Publ. Protein Soc.* **2019**, *28*, 454–463, doi:10.1002/pro.3528.
5. Hajizadeh, N.R.; Franke, D.; Jeffries, C.M.; Svergun, D.I. Consensus Bayesian Assessment of Protein Molecular Mass from Solution X-Ray Scattering Data. *Sci. Rep.* **2018**, *8*, 7204, doi:10.1038/s41598-018-25355-2.
6. Franke, D.; Jeffries, C.M.; Svergun, D.I. Machine Learning Methods for X-Ray Scattering Data Analysis from Biomacromolecular Solutions. *Biophys. J.* **2018**, *114*, 2485–2492, doi:10.1016/j.bpj.2018.04.018.
7. Kabsch, W. XDS. *Acta Crystallogr. D Biol. Crystallogr.* **2010**, *66*, 125–132, doi:10.1107/S0907444909047337.
